# Hematological and inflammatory markers in Han Chinese patients with drug-free schizophrenia: relationship with symptom severity

**DOI:** 10.3389/fimmu.2024.1337103

**Published:** 2024-01-30

**Authors:** Cheng Yang, Yinghan Tian, Xiaoxue Yang, Lewei Liu, Chen Ling, Lei Xia, Huanzhong Liu

**Affiliations:** ^1^ Department of Psychiatry, School of Mental Health and Psychological Sciences, Anhui Medical University, Hefei, China; ^2^ Department of Psychiatry, Chaohu Hospital of Anhui Medical University, Hefei, China; ^3^ Department of Psychiatry, Anhui Psychiatric Center, Anhui Medical University, Hefei, China

**Keywords:** neutrophil-to-lymphocyte ratio, monocyte-to-lymphocyte ratio, schizophrenia, symptom severity, drug-free

## Abstract

**Background:**

There is a growing amount of evidence suggesting that immunity and inflammation play an important role in the pathophysiology of schizophrenia. In this study, we aimed to examine the relationship between hematological and inflammatory markers with symptom severity in Han Chinese patients with drug-free schizophrenia.

**Methods:**

This retrospective study was conducted at Chaohu Hospital of Anhui Medical University and data were extracted from the electronic medical record system over a 5-year period (May 2017 to April 2022), including participants’ general and clinical information as well as Brief Psychiatric Rating Scale (BPRS) scores and hematological parameters.

**Results:**

A total of 2,899 patients with schizophrenia were identified through the initial search. After screening, 91 patients and 141 healthy controls (HCs) were included. The patients had a higher value of neutrophils/lymphocytes ratio (NLR), monocyte/lymphocyte ratio (MLR), and platelet/lymphocyte ratio (PLR) than HCs (all *P* < 0.001). MLR was positively correlated with BPRS total score (*r* = 0.337, *P* = 0.001) and resistance subscale score (*r* = 0.350, *P* = 0.001). Binary logistic regression analyses revealed that severely ill was significantly associated with being male and a higher value of MLR (Natural Logaruthm, Ln) (all *P <* 0.05), and the receiver operating characteristic (ROC) analysis showed good performance of a regression model with an area under the curve (AUC) value of 0.787.

**Conclusion:**

Patients with drug-free schizophrenia have an unbalanced distribution of peripheral blood granulocytes, and elevated NLR, MLR and PLR. Patients with higher value of MLR tend to have more psychotic symptoms, especially those symptoms of hostility, uncooperativeness, and suspiciousness. Our study gives a preliminary indication that MLR is a potential predictor of disease severity in patients with drug-free schizophrenia.

## 1 Introduction

The inflammatory response may play an important role in the pathology of schizophrenia (1). Elevated levels of inflammation are frequently found in patients with schizophrenia, which might predict the disease prognosis and relapse (2, 3). The positive association was confirmed by the fact that psychiatric symptoms improve with the decrease in inflammation levels during treatment with antipsychotics (4).

Cytokines are key molecules that regulate inflammation, including pro-inflammatory cytokines and anti-inflammatory cytokines (5). Numerous studies have found that some pro-inflammatory and anti-inflammatory cytokines are elevated in the blood of patients with schizophrenia (6, 7). For example, a meta-analysis of cytokines in schizophrenia found higher levels of pro-inflammatory cytokines such as IL-6, TNF-α and sIL-2R in acute patients, and IL-6, IL-1β and sIL-2R in chronic patients than in healthy controls (8). It also found higher levels of anti-inflammatory cytokines IL-1RA and TGF-β in the unspecified (not acute or chronic) patients (8). Also, another meta-analysis with a large scale of 59 studies found first-episode patients (FEP) with schizophrenia have higher levels of IL-6 and TNF-α than the general population (9). As the inflammatory response occurs, activated cytokines can not only enter the brain through peripheral organs lacking the blood-brain barrier (BBB), but also induce the production of cytokines by cells that compose the BBB. Activated cytokines modulate dopaminergic neurotransmission directly or glutamatergic neurotransmitters indirectly through tryptophan metabolism (10–14). This action leads to brain dysfunction and ultimately to schizophrenia-like symptoms (15).

Since the physiological response of leukocytes under stressful conditions such as inflammation leads to an increase in the neutrophil count and a decrease in the lymphocyte count, the ratios between neutrophil and lymphocyte (NLR), monocyte and lymphocyte (MLR), and platelet and lymphocyte (PLR) are often used as inflammatory markers (16). NLR, MLR and PLR are more accessible than inflammatory cytokines in clinical practice and scientific research, which have been used to predict progression and prognosis of many non-psychiatric diseases, such as hepatocellular carcinoma, gastric cancer, autoimmune encephalitis etc. (17–19). In recent years, there has been an increase in studies of psychiatric disorders involving these inflammatory markers. A meta-analysis of eight observational studies showed that patients with psychotic disorders had significantly higher NLR, MLR and PLR compared to healthy controls (20). In previous studies, NLR was found to be positively associated with the severity of psychotic symptoms as measured by Positive and Negative Syndrome Scale (PANSS) and Clinical Global Impressions Severity (CGI-S) scale in patients with schizophrenia (21–23). Furthermore, Dawidowski et al. demonstrated that NLR decreased after treatment and could be an indicator of response to antipsychotic treatment (24).

However, inconsistent results have been reported in some other studies. For example, a large retrospective study in China found a significant decrease of PLR in patients with acute-onset schizophrenia compared with healthy controls (25). Two cross-sectional studies conducted in Turkey and a cohort study conducted in Europe showed that there was no correlation between NLR and psychotic symptom scores as measured by the BPRS or PANSS in patients with schizophrenia (26–28). Such differences may be attributed to the heterogeneity of subjects across studies (e.g., different ethnicities, antipsychotic intake, and periods of illness). Therefore, this current study aimed to evaluate (I) hematological and inflammatory markers (NLR, MLR and PLR) in Han Chinese patients with acute episode and drug-free schizophrenia; to explore (II) their correlations with total and subscale scores of BPRS; and (III) their potential associations with symptom severity in a predictive model.

## 2 Methods

### 2.1 Study design and participants

This retrospective study extracted 5-year data (May 2017 - April 2022) from the electronic medical record system of Chaohu Hospital of Anhui Medical University, which have a total of 300 psychiatric beds, and serve more than 1 million people in Anhui Province, China. Participants were included if they fulfilled the following criteria: (I) Han Chinese, aged between 18 and 70 years; (II) with a diagnosis of schizophrenia according to the International Classification of Diseases (ICD-10) made by two independent senior psychiatrists; (III) drug-free defined as not taking any psychiatric medication before or off psychiatric medication for one month prior to blood sampling (23). During the same period, age- and sex-matched healthy controls (HCs) without personal or family history of mental illness were recruited from the medical screening center of Chaohu Hospital, which is the largest hospital in Chaohu, serving a population of about 700,000 people. All participants (patients and HCs) were excluded if they had somatic conditions that could alter the inflammatory state, such as acute or chronic infections (High-sensitivity C-reactive protein > 10mg/L), and immune-inflammatory diseases based on recorded diagnosis from the electronic medical record system; or they had blood diseases or were taking medications that would alter blood cell counts.

The Medical Ethics Committee of Chaohu Hospital of Anhui Medical University reviewed and approved the protocol of this retrospective study (No. 202210-kyxm-015).

### 2.2 Data collection measurements

#### 2.2.1 Demographic and clinical characteristics

We collected participants’ demographic and clinical information including age (years), sex (male/female), BMI (kg/m^2^) and duration of illness (months). BMI was calculated by the formula: weight (kg)/height (meters^2^).

#### 2.2.2 Laboratory measurements

Blood samples were collected between 06:00 and 08:00 AM after an overnight fast. Blood counts were measured by the Sysmex XN-9000 automated counter in the clinical laboratory department of Chaohu Hospital, including white blood cell (WBC), neutrophil, lymphocyte, monocyte, and platelet counts. NLR (neutrophils/lymphocytes ratio), MLR (monocyte/lymphocyte ratio), and PLR (platelet/lymphocyte) were calculated accordingly.

#### 2.2.3 Psychiatric symptoms assessment

Psychotic symptoms were assessed by Chinese version of the 18-item Brief Psychiatric Rating Scale (BPRS). All items were rated on a 7-point Likert scale ranging from 1 (not present) to 7 (extremely severe), with higher total scores indicating more severe symptoms (29). According to previous studies, the 18 items of the BPRS can be divided into the five subscales of affect, positive symptoms, negative symptoms, resistance, and activation (30). In this study, a cut-off value of 53 for the BPRS total score was used (mildly or moderately ill: 18-53; and severely ill: >53) (31).

### 2.3 Statistical analysis

Statistical analyses were performed using SPSS version 23.0 (SPSS IBM, Chicago, IL, USA). Demographic and clinical data are presented as mean (standard deviation, SD), and frequency distribution (%). For comparison between groups, independent samples t-test, Mann-Whitney *U* test, and Chi-square test were used as appropriate. When we compared the hematological and inflammatory markers between patients and HCs, analysis of covariance (ANCOVA) was used to control for potential confounding factors. Pearson (for normally distributed variables) or Spearman (for non-normally distributed variables) correlation analyses were used to examine the correlation between BPRS scores and hematologic parameters as well as inflammatory markers. Bonferroni corrections were applied to these analyses to adjust for multiple tests. Logarithmic conversions were performed for NLR and MLR in the logistic regression model. A logistic regression analysis (Forward Stepwise method) was used to examine the independent correlates associated with “severe illness” (yes or no), with variables that were significant (*P* < 0.05) in the univariate analyses as independent variables. We also conducted logistic regression analyses (Enter method) to examine the association between each hematological and inflammatory marker and “severe illness” after adjusting for age, gender, BMI, and duration of illness (**Supplementary Table 1**). Then, a receiver operating characteristic (ROC) curve was plotted and area under the curve (AUC) values were also calculated to assess the discriminatory power of the fitted model. The statistical significance was set at *P* < 0.05 (two-tailed).

## 3 Results

### 3.1 Case selection and participant characteristics

**Figure 1:**
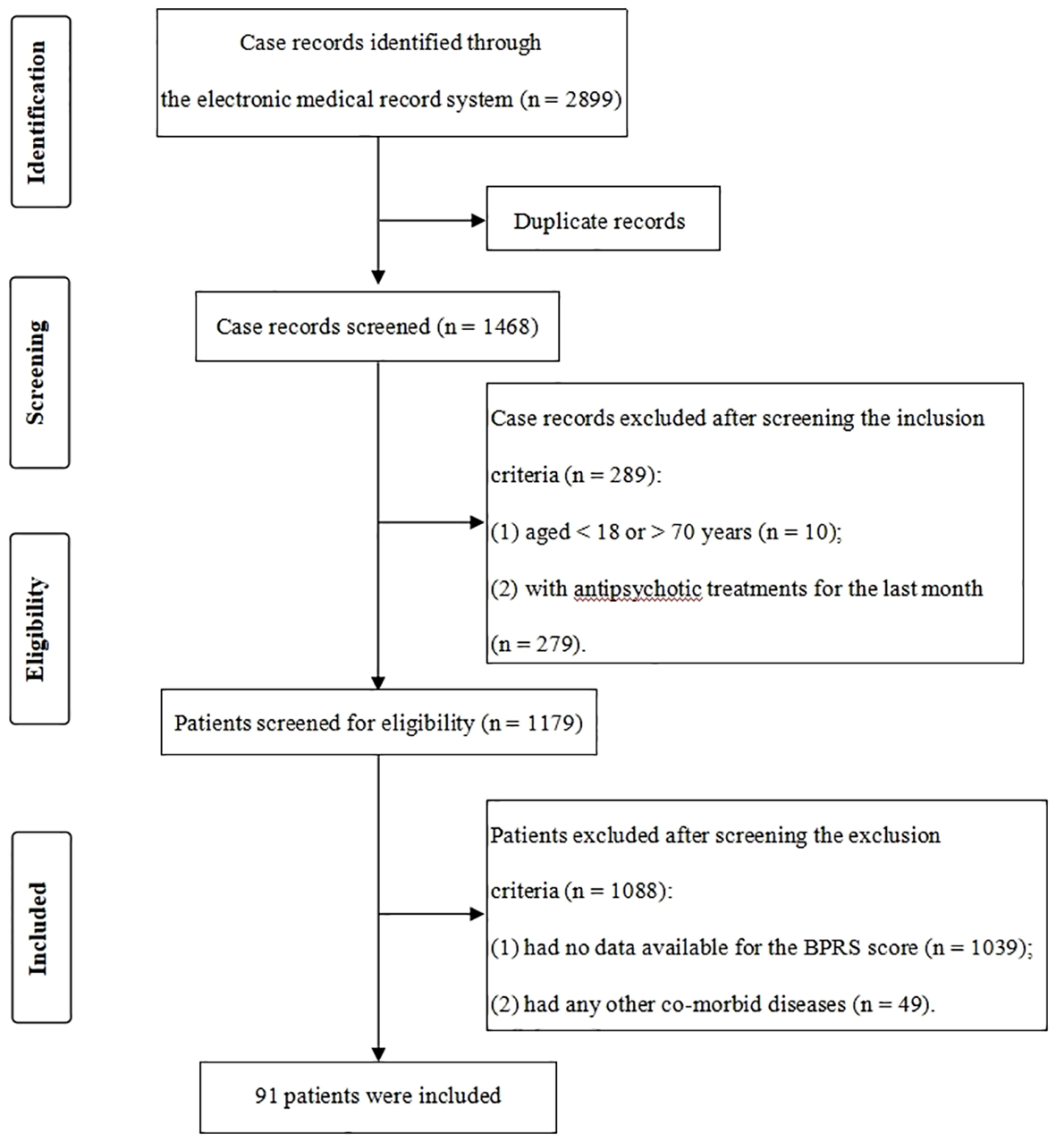
Flowchart of the case records selection. For patients with multiple admissions, only their first admission data were extracted for analyses.

We obtained 2,899 potentially relevant case records from the initial search (**Figure 1**). After duplicates excluded (n = 1,431) and screening according to the specific criteria (n = 1,377), 91 patients with drug-free schizophrenia were finally included. Meanwhile, 141 age- and sex-matched HCs from the local medical screening center were included. In this study, the mean age of patients and HCs was 39.27 ± 14.04 years and 41.52 ± 10.82 years, respectively, and 40.7% of patients and 46.8% of HCs were male. There were no significant differences in age, gender and BMI between groups (*P* > 0.05) (**Table 1**).

**Table 1:** Socio-demographic and clinical characteristics of the participants.

Variables	Patients (*n*=91)	Healthy controls (*n*=141)	*t/Z/χ2*	*P*
Age (years)	39.27 ± 14.04	41.51 ± 10.82	-1.203	0.229
Male (%)	37 (40.7)	66 (46.8)	0.847	0.357
BMI (kg/m2)	22.44 ± 1.96	22.68 ± 2.17	-0.715 ^a^	0.474
Duration of illness (months)	57.60 ± 86.54	–		
Hematological parameters				
WBC (k/μl)	7.08 ± 1.90	6.24 ± 1.36	-3.926 ^a^	**<0.001**
Neutrophil (k/μl)	4.78 ± 1.75	3.85 ± 1.14	-4.683 ^a^	**<0.001**
Lymphocyte (k/μl)	1.66 ± 0.65	1.86 ± 0.43	-4.250 ^a^	**<0.001**
Monocyte (k/μl)	0.53 ± 0.18	0.37 ± 0.10	-7.448 ^a^	**<0.001**
Platelets (k/μl)	214.98 ± 64.56	198.72 ± 44.11	-2.107	**0.037**
NLR	3.28 ± 1.70	2.16 ± 0.81	-5.647 ^a^	**<0.001**
MLR	0.36 ± 0.18	0.20 ± 0.06	-8.439 ^a^	**<0.001**
PLR	144.36 ± 68.36	111.52 ± 34.66	-4.133 ^a^	**<0.001**
BPRS total score	49.33± 10.11	–		
Affect subscale	7.95 ± 3.02	–		
Negative symptoms subscale	10.73 ± 3.17	–		
Positive symptoms subscale	12.86 ± 3.74	–		
Resistance subscale	5.90 ± 2.91	–		
Activation subscale	11.90 ± 4.41	–		

BMI, body mass index; WBC, white blood cell; NLR, neutrophil/lymphocyte ratio; MLR, monocyte/lymphocyte ratio; PLR, platelet/lymphocyte ratio; BPRS, Brief Psychiatric Rating Scale. ^a^Mann-Whitney U test. Bolded P values < 0.05.

### 3.2 Comparisons between patients and healthy controls

**Figure 2:**
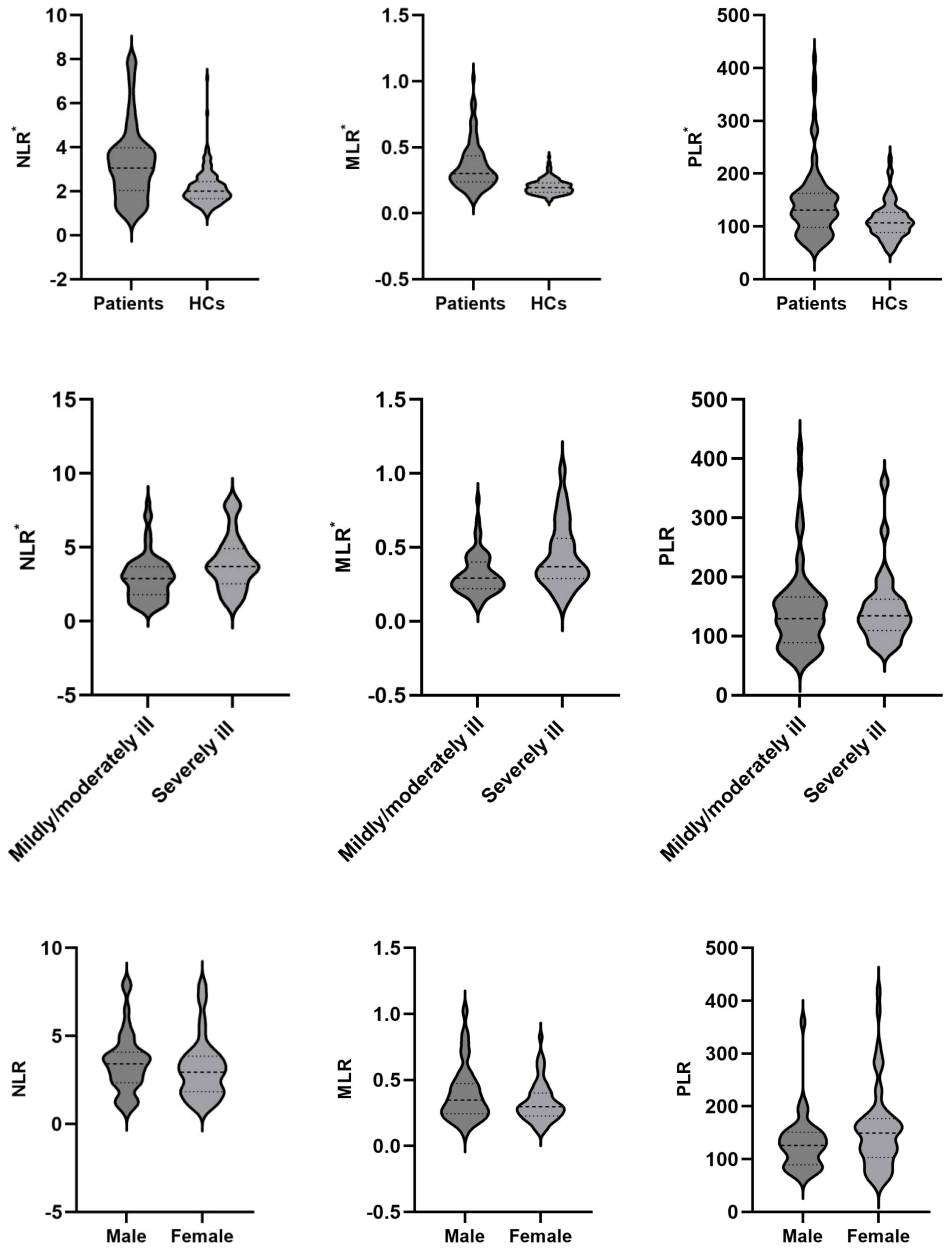
NLR, MLR and PLR between different groups. NLR, neutrophil/lymphocyte ratio; MLR, monocyte/lymphocyte ratio; PLR, platelet/lymphocyte ratio; *P < 0.05.

As shown in **Table 1**, the patients had a higher count of WBC, neutrophil, monocyte, and platelet, a lower count of lymphocyte, and higher values of NLR, MLR, and PLR than the HCs (all *P* < 0.05). ANCOVA showed that differences in the WBC, neutrophil, monocyte, platelet, lymphocyte, NLR, MLR, and PLR between the two groups remained significant after controlling for age, sex and BMI. The comparisons of NLR, MLR and PLR between different groups were shown in **Figure 2**.

### 3.3 Correlations between BPRS scores with hematological and inflammatory markers in patients

The total score of BPRS was positively correlated with the counts of WBC (*r* = 0.250, *p* = 0.017), neutrophil (*r* = 0.337, *P* = 0.001) and monocyte (*r* = 0.331, *P* = 0.001), and the value of NLR (*r* = 0.285, *P* = 0.006) and MLR (*r* = 0.337, *P* = 0.001). After Bonferroni correction (α = 0.05/48 = 0.001), the correlations between the total score of BPRS with neutrophils, monocytes and MLR remained significant. The correlations between the subscale scores of BPRS with hematological parameters were shown in **Table 2**. The scatterplots of the correlations between total and subscale scores of BPRS and MLR (Ln) were shown in **Supplementary Figure S1**.

**Table 2:** Correlations between BPRS scores with hematological and inflammatory markers.

Variables	BPRS total score		BPRS affect score		BPRS negative symptom score		BPRS positive symptom score		BPRS activitate score		BPRS resistance score	
	*r*	*P*	*r*	*P*	*r*	*P*	*r*	*P*	*r*	*P*	*r*	*P*
WBC (k/μl)	0.250	**0.017**	-0.170	0.108	0.226	**0.031**	0.141	0.184	0.287	**0.006**	0.161	0.127
Neutrophil (k/μl)	0.337	0.001*	-0.130	0.219	0.241	**0.021**	0.189	0.072	0.265	**0.011**	0.279	**0.007**
Lymphocyte (k/μl)	-0.075	0.477	-0.061	0.567	0.083	0.436	-0.013	0.901	0.038	0.718	-0.191	0.070
Monocyte (k/μl)	0.331	0.001*	-0.170	0.106	0.223	**0.034**	0.240	**0.022**	0.363	<0.001*	0.235	**0.025**
Platelets (k/μl)	-0.107	0.314	0.044	0.678	-0.082	0.437	-0.019	0.859	0.053	0.618	-0.176	0.094
NLR	0.285	**0.006**	-0.046	0.667	0.101	0.342	0.127	0.229	0.138	0.191	0.325	**0.002**
MLR	0.337	0.001*	-0.060	0.572	0.096	0.366	0.213	**0.043**	0.235	**0.025**	0.350	0.001*
PLR	0.036	0.736	0.128	0.227	-0.073	0.494	0.044	0.679	-0.090	0.396	0.052	0.621

WBC, white blood cell; NLR, neutrophil/lymphocyte ratio; MLR, monocyte/lymphocyte ratio; PLR, platelet/lymphocyte ratio; BPRS, Brief Psychiatric Rating Scale. *P < 0.05/48 = 0.001 (Bonferroni correction). Bolded P values < 0.05.

Spearman correlation analyses were applied for all correlation analyses.

### 3.4 Factors associated with severely ill patients

The patients in severely ill group had a higher count of WBC, neutrophil and monocyte, and higher values of NLR and MLR than those in mildly/moderately ill group (all *P* < 0.05) (**Table 3**). Further binary logistic regression analysis (forward stepwise method) found that severely ill was significantly associated with being male (OR = 6.427, 95% CI = 2.292-18.024) and a higher value of MLR (Ln) (OR = 4.236, 95% CI = 1.297-13.837) (all *P* < 0.05) (**Table 4**).

**Table 3:** Socio-demographic and clinical characteristics of patients in mildly/moderately ill and severely ill group.

SVariables	Mildly/moderately ill (n=63)	Severely ill (n=28)	*t/Z/χ2*	*P*
Age (years)	38.76 ± 14.05	40.43 ± 14.21	-0.520	0.604
Male (%)	17 (26.98)	20 (71.43)	15.870	**<0.001**
BMI (kg/m2)	22.68 ± 2.44	22.52 ± 1.01	-0.448 ^a^	0.654
Duration of illness (months)	57.37 ± 71.38	62.94 ± 117.10	-0.771 ^a^	0.441
WBC (k/μl)	6.78 ± 1.68	7.77 ± 2.21	-2.358	**0.021**
Neutrophil (k/μl)	4.44 ± 1.49	5.55 ± 2.07	-2.887	**0.005**
Lymphocyte (k/μl)	1.73 ± 0.72	1.51 ± 0.41	-0.993 ^a^	0.321
Monocyte (k/μl)	0.49 ± 0.15	0.61 ± 0.22	-2.469 ^a^	**0.014**
Plt (k/μl)	218.25 ± 67.92	207.61 ± 56.73	0.724	0.471
NLR	2.99 ± 1.54	3.98 ± 1.86	-2.674 ^a^	**0.007**
MLR	0.32 ± 0.14	0.44 ± 0.22	-2.773 ^a^	**0.006**
PLR	142.94 ± 71.66	146.33 ± 60.29	-0.537 ^a^	0.591

BMI, body mass index; WBC, white blood cell; Plt, platelets; NLR, neutrophil/lymphocyte ratio; MLR, monocyte/lymphocyte ratio; PLR, platelet/lymphocyte ratio; BPRS, Brief Psychiatric Rating Scale. ^a^Mann-Whitney U test. Bolded P values < 0.05.

**Table 4:** Independent factors associated with severely ill in patients.

Variables	P	OR	95%CI	
			Lower	Upper
Male (ref. female)	**<0.001**	6.427	2.292	18.024
MLR (Ln)	**0.017**	4.236	1.297	13.837

MLR, monocyte/lymphocyte ratio; OR, odds ratio; CI, confidence interval; Ref, reference group; Ln, Natural Logaruthm. Bolded P values < 0.05.

### 3.5 Receiver operating characteristic curves for the efficacy of the regression model

**Figure 3:**
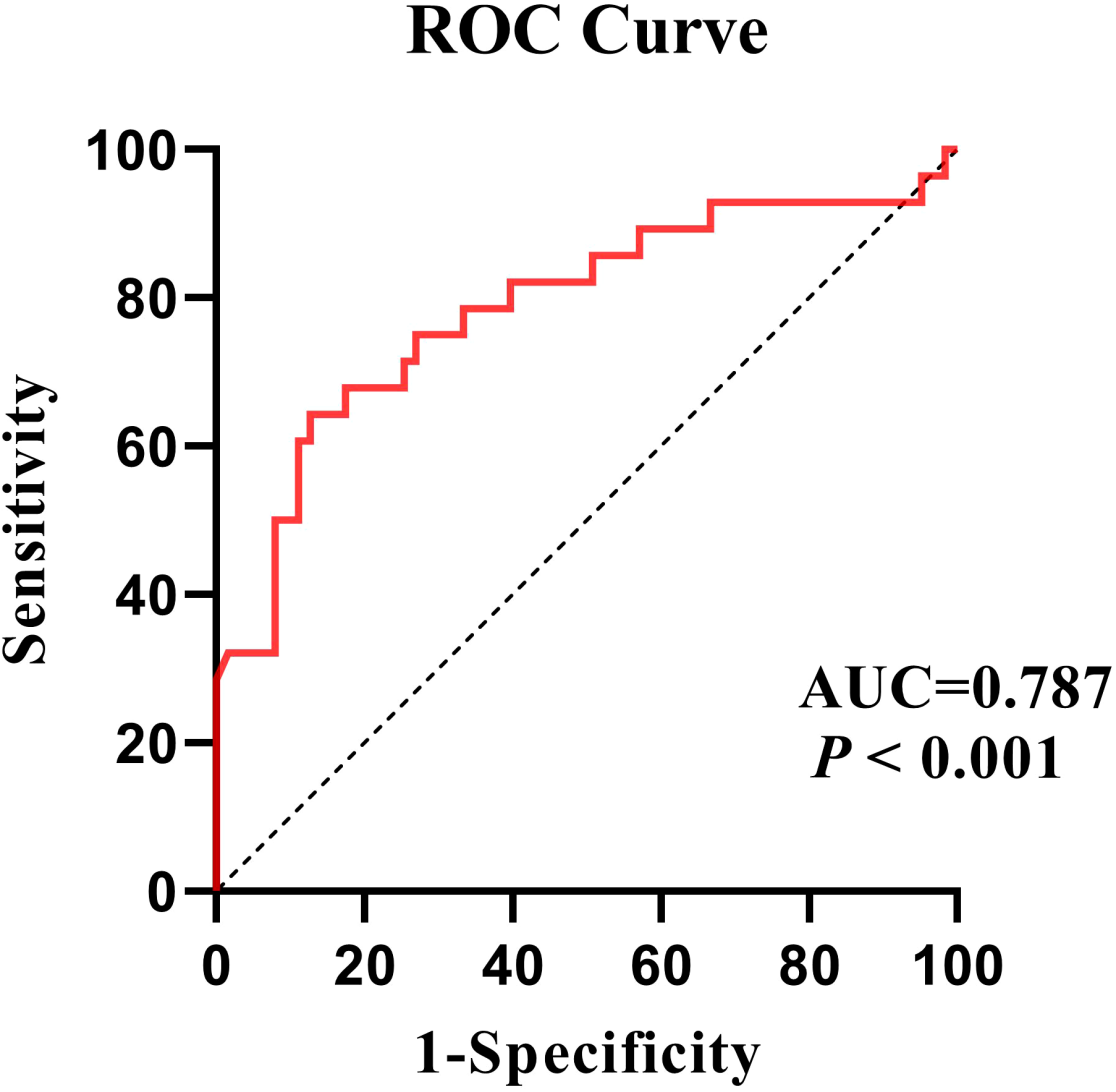
Using the severity of illness as a classification variable, ROC curves for the efficacy of the regression model.

The ROC analysis indicates a good discriminatory ability with a AUC value of 0.787 (95% CI: 0.674-0.899). The cut-off point was 0.443, with a sensitivity of 64.3% and a specificity of 87.3% (**Figure 3**).

## 4 Discussion

In this study, we explored the changes of hematological parameters and inflammatory markers (NLR, MLR and PLR), and their associations with symptom severity in Han Chinese patients with drug-free schizophrenia. First, we found elevated and unbalanced distribution of peripheral blood leukocytes in these patients, characterized by elevated neutrophil and monocyte counts and decreased lymphocyte counts. Similarly, in a cohort study, elevated neutrophil and monocyte counts were found in drug-naïve FEP and unmedicated patients with schizophrenia at baseline (32). The mechanism of such changes may be related to the deficiency in immunoregulation mediated by the endocannabinoid system in patients with schizophrenia. A study conducted a network analysis to assess the association between cannabinoid receptor expression on immune response-associated cells and cytokine levels in patients with schizophrenia and found that patients with schizophrenia had higher expression of cannabinoid receptors and a simpler network of correlations with cytokines compared to controls (33). Recent genome-wide association studies found genetically determined Immune abnormalities in patients with schizophrenia and identified shared genetic variants between schizophrenia and WBC counts (34). While, a meta-analysis of hematological parameters in patients with schizophrenia found that there were no significant differences in lymphocyte counts between patients (not limited to unmedicated patients) and the general population (35), which may be due to the heterogeneity of the participants across studies. Second, we also found that platelet levels were higher in patients who had not taken medication during the prior month than in healthy controls. A retrospective investigation in India found a decrease in platelet levels in patients with schizophrenia following clozapine administration (36). These findings suggest that platelet counts in the circulation are susceptible to the antipsychotics taken. As calculated by the counts of these blood cells, the levels of inflammatory markers (NLR, MLR, and PLR) were higher in patients with drug-free schizophrenia than in HCs in this study, which is consistent with the results of most previous studies. For example, a cross-sectional study conducted in China found elevated NLR, MLR and PLR in first-episode patients with schizophrenia (37). Furthermore, Bustan et al. found that NLR was elevated in acute psychotic states and relatively declined during periods of clinical remission in adolescents with non-affective psychosis (38).

In addition, the present study found a positive association between these peripheral blood inflammatory markers and psychopathology in patients with drug-free schizophrenia. The total score of BPRS was positively associated with the counts of WBC, neutrophil and monocyte, and the value of NLR and MLR. In fact, there is a possible vicious circle of interactions between schizophrenia and immune-inflammatory dysregulation. Increased levels of inflammation may be closely intertwined with the onset of schizophrenia and exacerbation of psychotic symptoms. A cross-sectional study conducted in China found that NLR was positively associated with the CGI-S score and the BPRS total score in drug-free patients (23). In addition, a retrospective study found that there were significant differences in MLR between three groups (relapse schizophrenia > remission schizophrenia = healthy control), suggesting that MLR may be a state marker for schizophrenia (39). Although the mechanisms underlying these associations are complex, the immune-inflammatory hypothesis of schizophrenia may provide a partial explanation. Since NLR and MLR are calculated as neutrophils/lymphocytes and monocytes/lymphocytes, they reflect discuss neutrophil- and monocyte-mediated immune responses (innate immune response) as well as lymphocyte-mediated immune responses (adaptive immune response), which are possibly related to the manifestation of schizophrenia symptoms.

First, the BBB appears to be disrupted in schizophrenia (40). Neutrophils directed by specific chemokines can infiltrate the brain through the damaged BBB (41), which may lead to brain tissue damage and more psychotic symptoms in schizophrenia. Neuroimaging studies found that higher blood neutrophil levels are associated with gray matter reduction and ventricular enlargement in patients with first-episode schizophrenia (42). A clinical study also found peripheral blood neutrophil counts were significantly and positively correlated with PANSS total and positive subscale scores in patients with schizophrenia (22). Second, the monocyte macrophage system is also a critical component of innate immunity and plays an important role in the immune-inflammatory hypothesis of schizophrenia. Increased peripheral monocytes may suggest activation of microglia in central nervous system (43). As a type of macrophage, activated microglia interferes with neuronal development and disrupts the function of neuronal circuits in the brain (44).For example, Cui et al. (45) found that high transcript levels of monocyte genes across cortical regions were associated with cortical thinning in patients with psychosis by a high-resolution T1-weighted structural image, while cortical thickness could reflect neurodevelopmental status. Third, our study found that lymphocytes significantly decreased in patients but did not correlate with the total score of BPRS. Our interpretation was that adaptive immunity may be involved in the pathophysiological mechanisms of schizophrenia, but its action may be related to the activation phenotype of lymphocytes and the subtype of schizophrenia. For example, a meta-analysis reported that an increased number of CD56-positive cells are important markers of disease exacerbation in schizophrenia (46). Autopsy studies from post-mortem brain samples found that T and B cell numbers were increased in the hippocampus of patients with schizophrenia, especially those with predominantly negative symptoms (47). Therefore, we think it is necessary to stratify lymphocyte activation phenotypes and schizophrenia subtype for investigating the pathophysiological role of lymphocytes in schizophrenia.

Regarding the BPRS subscales, we found that NLR was positively correlated with the resistance subscale score, and MLR was positively correlated with the positive symptoms, activitate, and resistance subscale scores. However, only the correlation between MLR and resistance subscale score remained significant after Bonferroni correction. To the best of our knowledge, this is the first study revealing a correlation between MLR and resistance subscale scores in patients with drug-free schizophrenia. Resistance is defined as hostility, uncooperativeness and suspiciousness in patients with schizophrenia. A cross-sectional study found that higher level of IL-1β was associated with higher resistance subscale scores in patients with first-episode schizophrenia (48). Since IL-1β is produced by monocytes, and the higher level of IL-1β often reflects activation of the monocyte-macrophage system, which is equivalent to elevated value of MLR (monocyte count as numerator) to some extent (49). Therefore, in clinical practice, it is necessary to monitor the MLR, which could contribute to a comprehensive assessment of psychotic symptoms in patients with schizophrenia, especially those with hostility, uncooperativeness, and suspiciousness.

We further divided the patients into two groups of mildly/moderately or severely ill, and found that severely ill was significantly associated with being male and a higher value of MLR (Ln). Clinical studies have shown that male patients with schizophrenia tend to have a higher score of BPRS or PANSS, so they are more inclined to be defined as being severely ill (50, 51). This may be attributed to the differences in brain structure and function, neurodevelopment, and psychosocial factors between males and females (52). For example, a clinical study found differences in brain functional alterations between male and female patients with schizophrenia and were correlated with a series of psychotic symptoms (53). An animal study found that there were sex differences in dopaminergic projections from the midbrain to the basolateral amygdala in mice that were associated with schizophrenia-like behaviors (54). In addition, there is an “estrogen-protective” effect in female patients with schizophrenia (55). A randomized placebo-controlled trials found that estradiol are effective and safe adjunctive treatments to improve schizophrenia symptoms for female patients (56). Sex differences in immune cells and their signaling in the adult brain have been found to be associated with many diseases with altered brain development or plasticity, such as schizophrenia (57). However, there were no differences in inflammatory markers (NLR, MLR and PLR) between male and female patients in this study.

Although NLR, MLR, PLR, and other inflammatory markers may have potential diagnostic value in distinguishing schizophrenia from the general population, their specificity for schizophrenia is low because they are also elevated in many other psychiatric disorders (58, 59). In this cross-sectional study, we have a preliminary finding that the MLR may has a good predictive power on whether or not being severely ill in patients with schizophrenia. Specifically, the ROC analysis showed good performance of a regression model with an AUC value of 0.787. Thus, MLR is a potential biomarker for predicting the symptom severity in drug-free patients with schizophrenia. This finding provides a new insight that monitoring the MLR may allow for an objective assessment of the efficacy of antipsychotic treatment. Moreover, a meta-analysis revealed that some agents with anti-inflammatory properties such as aspirin, minocycline, and N-acetylcysteine have an adjunctive efficacy for schizophrenia, especially for first-episode psychosis and early-phase schizophrenia (60). These findings illustrate the importance of monitoring and intervening against immune-inflammatory abnormalities, which probably play a critical role in the pathogenesis, diagnosis, and treatment in early-phase or untreated schizophrenia.

Several limitations in this study should be noted. First, because this was a retrospective cross-sectional study, we were not able to address the direction of causality between inflammatory markers with symptom severity. Second, the study initially screened more than 2,000 cases over a five-year period, yet all of the cases were from a single center, with a weak representativeness. Third, it were not taken into account the fact that “drug-free” patients may receive psychotherapy and counseling intervention, which people often use to prevent or alleviate symptoms at the onset. Finally, relevant factors which may be associated with inflammation in patients with schizophrenia, such as lifestyle habits (smoking, drinking and exercise condition) and other clinical symptoms (sleep condition), were not examined.

## 5 Conclusion

In conclusion, patients with drug-free schizophrenia have an unbalanced distribution of peripheral blood granulocytes, and elevated NLR, MLR and PLR compared to healthy controls. Patients with higher value of MLR tend to have more psychotic symptoms, especially those symptoms of hostility, uncooperativeness, and suspiciousness. Our results also revealed that MLR is a potential marker for predicting the severity of illness in drug-free patients with drug-free schizophrenia. Given the accessibility in clinical practice, monitoring the MLR allows for a comprehensive assessment of psychotic symptoms in schizophrenic patients. However, as the findings of this study have not been generalized to all subtypes of schizophrenic patients, the relationship between MLR and psychopathology in schizophrenia needs to be treated with caution due to the heterogeneity of subjects across studies.

## Data availability statement

The original contributions presented in the study are included in the article/**Supplementary Material**. Further inquiries can be directed to the corresponding authors.

## Ethics statement

The studies involving humans were approved by Chaohu hospital of Anhui medical university. The studies were conducted in accordance with the local legislation and institutional requirements. The participants provided their written informed consent to participate in this study.

## Author contributions

CY: Writing – original draft, Writing – review & editing. YT: Writing – review & editing. XY: Writing – review & editing. LL: Writing – review & editing. CL: Writing – review & editing. LX: Writing – review & editing. HL: Funding acquisition, Writing – review & editing.
