# Supplementary material for: Hematological and inflammatory markers in Han Chinese patients with drug-free schizophrenia: relationship with symptom severity
Source: Front Immunol. 2024 Jan 30;15:1337103. doi: 10.3389/fimmu.2024.1337103 (PMC10861680; doi:10.3389/fimmu.2024.1337103)
Supplement: Supplementary file 1 [file DataSheet_1.docx]

**Supplementary Appendix**

**Supplementary Table 1. Associations between hematological markers and “severe illness” in patients after adjusting for age, gender, BMI, and duration of illness.**

| **Variables** | ***P*** | **OR** | **95% CI** | |
| --- | --- | --- | --- | --- |
|  |  |  | **Lower** | **Upper** |
| **WBC** |  |  |  |  |
| Age (years) | 0.139 | 1.032 | 0.990 | 1.075 |
| Male (ref. female) | **<0.001** | 6.788 | 2.340 | 19.691 |
| BMI (kg/m^2^) | 0.218 | 0.842 | 0.640 | 1.107 |
| Duration of illness (months) | 0.575 | 1.002 | 0.996 | 1.007 |
| WBC (k/μl) | 0.067 | 1.352 | 0.979 | 1.867 |
| **Neutrophil** |  |  |  |  |
| Age (years) | 0.141 | 1.031 | 0.990 | 1.075 |
| Male (ref. female) | **<0.001** | 7.101 | 2.410 | 20.921 |
| BMI (kg/m^2^) | 0.169 | 0.820 | 0.619 | 1.088 |
| Duration of illness (months) | 0.575 | 1.002 | 0.996 | 1.007 |
| Neutrophil (k/μl) | **0.018** | 1.533 | 1.075 | 2.187 |
| **Monocyte** |  |  |  |  |
| Age (years) | 0.241 | 1.024 | 0.984 | 1.064 |
| Male (ref. female) | **0.001** | 6.591 | 2.246 | 19.348 |
| BMI (kg/m^2^) | 0.299 | 0.871 | 0.672 | 1.130 |
| Duration of illness (months) | 0.544 | 1.002 | 0.996 | 1.008 |
| Monocyte (k/μl) | **0.040** | 20.887 | 1.154 | 377.924 |
| **NLR** |  |  |  |  |
| Age (years) | 0.335 | 1.020 | 0.980 | 1.061 |
| Male (ref. female) | **<0.001** | 8.278 | 2.764 | 24.790 |
| BMI (kg/m^2^) | 0.242 | 0.848 | 0.644 | 1.117 |
| Duration of illness (months) | 0.448 | 1.002 | 0.997 | 1.008 |
| NLR (Ln) | **0.017** | 3.817 | 1.273 | 11.448 |
| **MLR** |  |  |  |  |
| Age (years) | 0.439 | 1.016 | 0.977 | 1.056 |
| Male (ref. female) | **<0.001** | 7.574 | 2.551 | 22.481 |
| BMI (kg/m^2^) | 0.331 | 0.877 | 0.673 | 1.143 |
| Duration of illness (months) | 0.478 | 1.002 | 0.996 | 1.008 |
| MLR (Ln) | **0.020** | 4.248 | 1.259 | 14.329 |
| NLR: neutrophil/lymphocyte ratio; MLR: monocyte/lymphocyte ratio; OR: odds ratio; CI: confidence interval; Ref: reference group; Ln: Natural Logaruthm. Bolded *P* values < 0.05. | | | | |

**
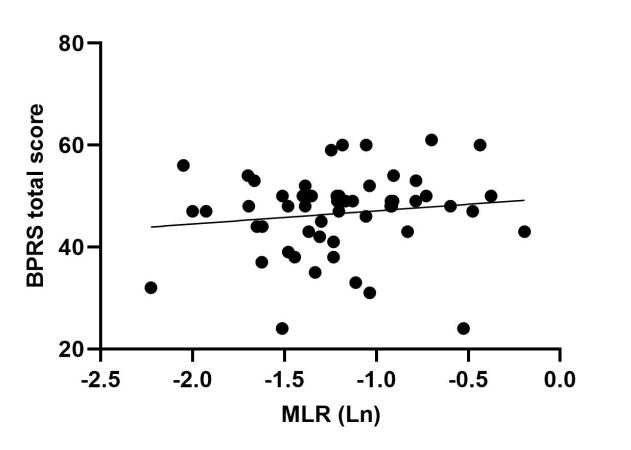

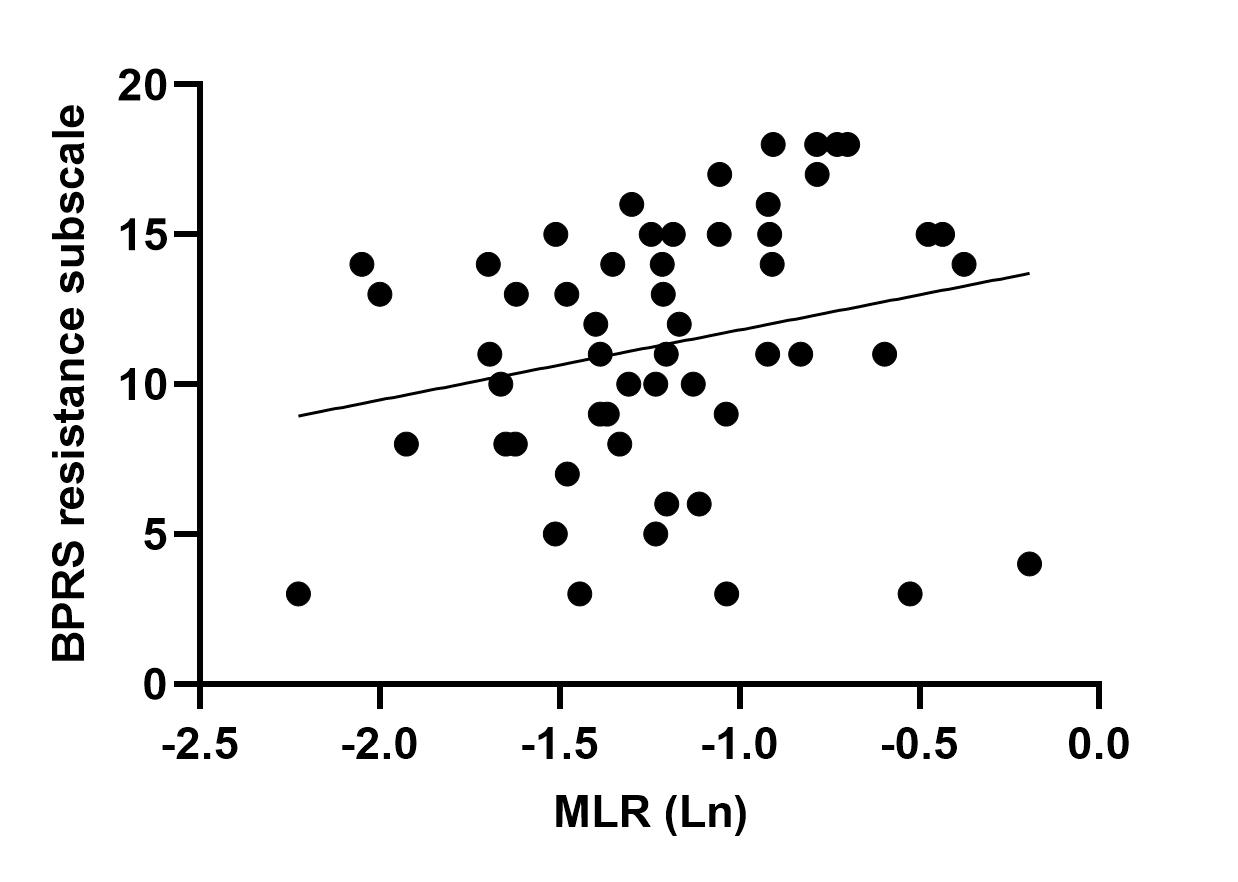
**

**Figure S1. Scatterplots of the correlations between total and subscale scores of BPRS and MLR (Ln).**

MLR: monocyte/lymphocyte ratio; Ln: Natural Logaruthm
